# Association between asthma with dry eye disease: A protocol for systematic review and meta-analysis

**DOI:** 10.1097/MD.0000000000022519

**Published:** 2020-10-09

**Authors:** Qun Huang, Yang Yang, Tingting Liao, Chuantao Zhang, Yanlin Zheng, Wanjie Wang, Xili Xiao, Jing Wang, Juan Wang

**Affiliations:** a Department of Ophthalmology; b Department of Respiratory Medicine; c Department of endocrinology, Hospital of Chengdu University of Traditional Chinese Medicine, Chengdu, China.

**Keywords:** asthma, dry eye disease, meta-analysis, protocol

## Abstract

**Backgoun:**

Asthma and dry eye disease are common clinical diseases. Studies have shown that asthma is related to dry eye, but there is no high-quality evidence-based medical evidence.

**Method:**

This protocol and final study will be conducted in accordance with the Preferred Reporting Items for Systematic review and Meta-Analysis Protocols 2015 statement. We will search PubMed, EMBASE, ISI Web of Science, China National Knowledge Infrastructure for all relevant literature published from their inception up to August 1, 2020. Literature search, data extraction, and quality assessment will be carried out independently by two researchers, and a third researcher will resolve differences when necessary. The association between dry eye disease and asthma will indicate as odds ratio with 95% confidence interval and statistically analyzed using RevMan 5.3 software. If the studies included have high heterogeneity, we will conduct sensitivity analysis and subgroup analysis.

**Results:**

The protocol is intended to guide a meta-analysis aimed at identifying and quantifying the association between asthma and dry eye disease.

## 1 Introduction

Dry eye disease (DED) is a multifactorial ocular surface disease characterized by loss of tear film homeostasis, with clinical symptoms including ocular discomfort and blurred vision.^[1]^ Because of its special form of disease, it not only causes a huge economic burden, but also seriously affects the quality of life and work efficiency of patients.^[2,3]^ The incidence of dry eye is related to age and sex, but its specific pathogenesis is not fully understood.^[4,5]^

Asthma is a common chronic respiratory disease characterized by inflammation, airway remodeling and hyperresponsiveness.^[6]^ Patients with asthma often suffer from other allergic diseases, including allergic rhinitis, allergic conjunctivitis, and atopic dermatitis.^[7,8]^ Recent studies have shown that allergic diseases, asthma, a history of arthritis, gout, use of corticosteroids, antidepressants, and hormone replacement therapy may be risk factors for DED.^[9,10]^

Although a number of studies have investigated the link between asthma and DED, the findings are inconsistent^[11,12]^ and there is a lack of high-quality evidence-based medical evidence. Therefore, we will collect relevant literature and conduct a meta-analysis to assess the association between asthma and DED.

## 2 Objective

The purpose of this study is to evaluate the association between asthma and DED through a meta-analysis of published data.

## 3 Methods

### 3.1 Study registration

The protocol has been registered in the Open Science Framework (OSF) (registration number: DOI 10.17605/OSF.IO/UHN38). This systematic review and meta-analysis will be reported inaccordance with the Preferred reporting items for systematic review and meta-analysis protocols (PRISMA-P) 2015 statement.^[13]^ Ethical approval is not required for the study.

### 3.2 Literature search strategy

According to the search strategy proposed by Cochrane, 2 researchers will independently search multiple electronic databases, including PubMed, EMBASE, ISI Web of Science, and China National Knowledge Infrastructure to retrieve all eligible articles published from their inception up to August 1, 2020, without any language restriction. The English database will use a combination of Medical Subject Headings (MeSH) alongside free terms to search for potentially qualified publications (Table 1). The following terms will be utilized (“Asthma”or “Asthmas” or “Bronchial Asthma” or “Asthma, Bronchial”) and (“Dry Eye Syndromes” or “Dry Eye Syndrome” or “Dry Eye Disease” or “Dry Eye Diseases” or “Dry Eye” or “Dry Eyes” or “Evaporative Dry Eye Disease” or “Evaporative Dry Eye Syndrome” or “Evaporative Dry Eye” or “Dry Eye, Evaporative” or “Evaporative Dry Eyes”). We will also manually screen the reference lists of original and review articles for additionally eligible studies.

**Table 1:**
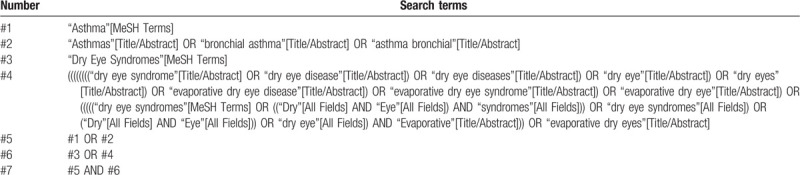
PubMed search strategy.

### 3.3 Inclusion criteria

#### 3.3.1 Types of participants

The diagnosis of asthma and DED in the study group is based on well-established criteria or according to a clinical diagnosis made by clinical physicians. The control subjects should be free of any history of asthma or DED. No restrictions will be placed on age, sex, or country.

#### 3.3.2 Types of studies

Types of study are observational studies including cross-sectional studies, cohort studies, case–control studies, or epidemiological studies; The main outcome is the association between DED and asthma, as indicated by odds ratio (OR) and its 95% confidence interval (CI), which should be either provided directly in the original article or could be calculated based on the original data.

### 3.4 Exclusion criteria

Abstracts, editorial letters, reviews, case reports, book chapters, and organizational guidelines were excluded from the present analysis. If studies with overlapping participants were encountered, the reports with the largest sample and the most recent reports were included in the present meta-analysis. If no data are available in the original article, the corresponding author of relevant study would be contacted via email. If the corresponding author did not response after we sent 3 e-mails, this article would not be used for quantitative synthesis.

### 3.5 Selection of studies and data extraction

**Figure 1:**
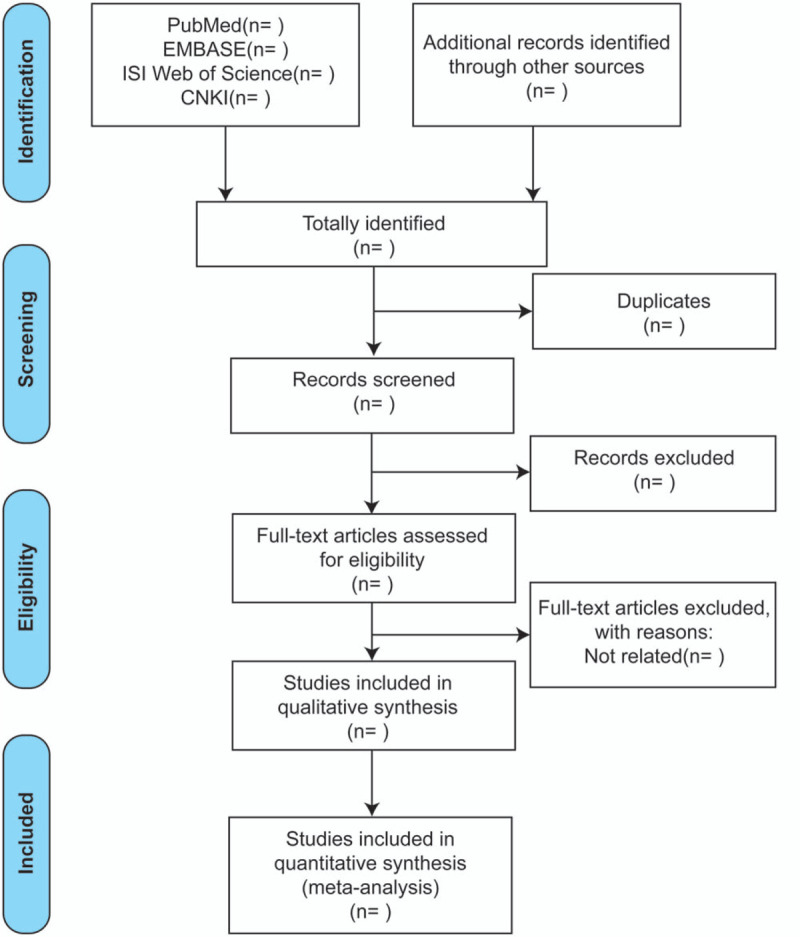
Flow chart of study selection. CNKI = China National Knowledge Infrastructure.

Two reviewers (XX and JW) will independently review all the included literature, extract data, and cross-check. If there are differences of opinion, the third reviewer (YZ) will negotiate and resolve. In the literature screening, the title of the text is read first. After the obviously irrelevant literature is excluded, the abstract and full text are further read to determine whether to include. A flow chart of study selection is shown in Figure 1. Data extracted from all qualified articles include: first author, year of publication, country where the study was conducted, sample size, demographic characteristics of participants in different groups, strategies for confirmation of DED and asthma, adjustment of confounding factors for effect assessment.

### 3.6 Quality assessment

Two reviewers (WW and JW) will independently evaluate the quality of the included studies and a third reviewer (YZ) will be consulted for consensus if disagreement occurred. The Newcastle-Ottawa Scale was used to assess the risk of bias in case–control and cohort studies.^[14]^ The methodological quality of the cross-sectional studies was evaluated following the standards of the Agency for Healthcare Research and Quality.^[15]^

### 3.7 Statistical analysis and assessment of heterogeneity

RevMan 5.3 (Copenhagen: The Nordic Cochrane Center, The Cochrane Collaboration, 2014) will used for the meta-analysis and subgroup analysis. The association between asthma and DED was estimated using adjusted OR and unadjusted OR, expressed as a 95% CI. The confounding factors considered included age and sex. Before combing date from the included studies, statistical heterogeneity among studies for each outcome was estimated using a standard *χ*^2^ test and the Higgins *I*^2^ test (*P* > .1 and *I*^2^ < 50% indicated acceptable heterogeneity).^[16]^ A fixed model will be applied to estimate the pooled ORs if low heterogeneity among studies, otherwise the random model will be used.

### 3.8 Assessment of publication biases

The funnel plots will be utilized to analyze the potential publication bias if there are >10 studies. Begg rank correlation test and Egger linear regression test via Stata version 12.0 (Stata Corp LP) will be also used to evaluate the publication bias.

### 3.9 Sensitivity analysis and subgroup analysis

We will eliminate 1 study in turn and re-analyze to check the robustness and reliability of pooled outcome results. In the case of high heterogeneity, we will conduct subgroup analysis based on age, sex, and race.

### 3.10 Grading the quality of evidence

The quality of evidence for outcome will be assessed by the Grading of Recommendations Assessment, Development, and Evaluation (GRADE) working group approach. High, medium, low, or very low quality represents the 4 levels of evaluation.

## 4 Discussion

DED is a common clinical disease with a prevalence of 15% to 35%, which seriously affects the quality of life.^[17,18]^ Age is known to be an important factor, but the incidence of DED is still around 15% in people under 50 years of age.^[19]^ Therefore, the research of related risk factors is an important method, which helps us to explore the pathological mechanism and preventive treatment of DED. At present, many studies have proved the connection between asthma and dry eye. For example, through a comparative analysis, Dogru et al^[20]^ found that children with asthma have a higher rate of tear film instability, which may lead to future DED. Bielory^[21]^ found that antihistamines and anti-inflammatory drugs used to treat asthma and allergies may exacerbate DED by causing tear film dysfunction or conjunctival overreaction. However, there are also population-based studies showing no significant association between asthma and DED.^[11]^ Therefore, a comprehensive, systematic, and rational meta-analysis is necessary to assess the association between asthma and DED.

To our knowledge, this is the first systematic review and meta-analysis to evaluate the relationship between asthma and DED. However, as an observational meta-analysis, our study has some limitations. First of all, our research cannot reflect the causal relationship between asthma and DED. Secondly, the influence of environmental and drug factors cannot be ruled out. Finally, the lack of a universal “criterion standard” diagnosis of dry eye may have influenced our results. Nevertheless, our research will demonstrate and quantify the connection between asthma and dry eye, and provide references for clinical prevention and treatment.

## Author contributions

**Conceptualization:** Qun Huang, Chuantao Zhang.

**Investigation:** Xili Xiao, Jing Wang.

**Supervision:** Wanjie Wang, Juan Wang, Tingting Liao.

**Writing – original draft:** Yang Yang, Chuantao Zhang.

**Writing – review & editing:** Qun Huang, Yanlin Zheng.

## Abbreviations

Abbreviations: DED = dry eye disease, MeSH = Medical Subject Headings, NOS = Newcastle-Ottawa Scale, OR = odds ratio, PRISMA = Preferred Reporting Items for Systematic Review and Meta-Analyses.
